# Burden of Vitamin D, Vitamin B12 and Folic Acid Deficiencies in an Aging, Rural Indian Community

**DOI:** 10.3389/fpubh.2021.707036

**Published:** 2021-09-03

**Authors:** Jonas S. Sundarakumar, Shafeeq K. Shahul Hameed, SANSCOG Study Team, Vijayalakshmi Ravindranath

**Affiliations:** Centre for Brain Research, Indian Institute of Science, Bangalore, India

**Keywords:** vitamin D, vitamin B12, folic acid, deficiency, prevalence, burden, rural, Indians (India)

## Abstract

**Introduction:** The important role of micronutrient deficiencies in aging-related disorders including dementia is becoming increasingly evident. However, information on their burden in India is scarce, especially, among aging and rural communities.

**Methods:** Prevalence of vitamin D, B12 and folic acid deficiency was measured in an ongoing, aging cohort, from rural India–Srinivaspura Aging Neurosenescence and COGnition (SANSCOG) study cohort. Serum level estimation of vitamin D, B12 and folic acid, using chemiluminescence immunoassay, was performed on 1648 subjects (872 males, 776 females).

**Results:** Mean vitamin D, B12 and folic acid levels were 23.4 ± 10.6 ng/ml, 277.4 ± 194.4 pg/ml and 6 ± 3.5 ng/ml), respectively. Prevalence of low vitamin D (<30 ng/ml), vitamin D deficiency (<20 ng/ml), B12 deficiency (<200 pg/ml) and folic acid deficiency (<3 ng/ml) were 75.7, 39.1, 42.3, and 11.1%, respectively. Significantly more women had vitamin D deficiency, whereas more men had folic acid deficiency. Women belonging to the oldest age group (≥75 years) had the maximum burden of low vitamin D (94.3%) and folic acid deficiency (21.8%).

**Discussion:** Older, rural-dwelling Indians have high burden of vitamin D and B12 deficiencies, which is concerning given the potentially negative consequences on cognition, immunity and frailty in the aging population. Urgent public health strategies are needed to address this issue and prevent or mitigate adverse consequences.

## Introduction

Micronutrients, such as vitamin D, vitamin B12 and folic acid are essential for several important physiological functions (1–4). Deficiencies of these vitamins are known to be associated with a wide range of disorders, affecting nearly every system in the human body (5–10). In particular, the role of vitamin D (11–13), vitamin B12 and folic acid (14, 15) in aging and late-life cognition is increasingly recognized in recent times. Early detection of these micronutrient deficiencies along with appropriate public health interventions has the potential to positively impact various associated health conditions, including aging-related disorders.

Higher rates of malnutrition, poor health awareness and strained public healthcare systems including inadequate mass-screening or community-level interventions in low- and middle-income countries could potentially contribute to higher prevalence of micronutrient deficiencies. Wide variation in dietary patterns, according to geographical regions as well as religious and socio-cultural practices is another challenge in India. Food fortification as a public health strategy is not as prevalent in India as in developed countries barring a few exceptions, such as iodization of salt. Elderly Indians could be at higher risk of vitamin deficiencies, with the rural elderly likely to be even more vulnerable. Consequently, this group could be at increased risk for developing physical, mental and cognitive health problems.

Studies on the prevalence of vitamin D, vitamin B12 and folic acid deficiency in the Indian population are limited. Moreover, very few of these studies have focussed on the elderly age group. There are no large studies available on the prevalence of the above vitamin deficiencies among the elderly from rural India. Therefore, this study is significant as it would provide valuable insights into the burden of the above-mentioned micronutrient deficiencies in an aging, rural Indian community.

The Srinivaspura Aging Neurosenescence and COGnition (SANSCOG) study (16) is a unique, large-scale, prospective, community-based, aging cohort study that was started in rural India in 2017. This study recruits a cohort of consenting individuals aged 45 years and above, from the villages of Srinivaspura – a “taluk” or sub-district, located in Kolar district, in the southern Indian state of Karnataka. The aim of this study is to understand the diverse, longitudinal trajectories of aging as well as to identify the risk and protective factors for aging-related disorders, such as dementia. This is envisaged by conducting extensive, multimodal (clinical, cognitive, biochemical, genetic and neuroimaging) assessments in this rural Indian cohort, with long-term, periodic follow-up.

## Methods

### Study Population

This study sample includes 1,648 subjects (872 males and 776 females) from the SANSCOG cohort, who had completed their baseline clinical and cognitive assessments, and given blood samples for biochemical investigations. These participants were recruited into the SANSCOG study from the villages of Srinivaspura taluk, through an area sampling strategy. All subjects are from a rural background (hailing from villages within the administrative area of Srinivaspura taluk headquarters) and belong to a low socio-economic status. They are predominantly engaged in agriculture, with the majority being mango farmers (Srinivaspura is one of the largest mango-producing areas in India). Most of the subjects have low levels of formal education and have not witnessed significant lifestyle changes in the past few decades. Hence, this sample population is representative of the two-thirds of India's population (over 800 million) that live in more than 600,000 villages.

### Ethics Clearance and Informed Consent

The Institutional Ethics Committee of the Centre for Brain Research has given clearance to the SANSCOG study. Subjects gave voluntary, written informed consent for the study procedures including blood sample collection.

### Sample Collection

Overnight fasting blood samples were collected from the study subjects for a range of biochemical investigations. In view of the limited availability of public transport facilities in villages of our catchment area and in order to make it convenient for our participants, periodic blood collection camps were conducted in the participants' villages itself. Peripheral venous blood (15 ml total volume) was collected from each subject by trained phlebotomists, for biochemical, hematological and genetic tests as well as for biobank storage. This was then segregated into 5 tubes (4.5 ml each in 2 serum tubes, 1 ml in fluoride tube, 1.5 ml and 3.5 ml each in EDTA tubes). Blood collected in the serum gel tubes (for biochemical investigations) was centrifuged at 2,000 rpm for 10 min for serum separation at the blood collection site itself. Biochemical tests (including estimation of vitamin D, vitamin B12 and folic acid levels) were performed at the Central Diagnostic Laboratory Services, RL Jalappa Hospital and Research Centre, affiliated to Sri Devaraj Urs Academy of Higher Education and Research (SDUAHER), Kolar. This laboratory is accredited by the National Accreditation Board for Testing and Calibration Laboratories (NABL), India.

### Bioassays

Estimation of vitamin D, B12 and folic acid levels in serum was performed using chemiluminescence immunoassays on VITROS ECiQ Immunodiagnostic Systems (Ortho Clinical Diagnostics) using Intellicheck^®^ Technology. For vitamin D, levels of the metabolite 25-OH vitamin D were measured since this is the most reliable clinical indicator of vitamin D status (17). VITROS 25-OH vitamin D total reagent pack and VITROS 25-OH vitamin D total calibrators were used. For vitamin B12, VITROS vitamin B12 reagent packs 1/2, VITROS vitamin B12/folate reagent pack 3 and VITROS vitamin B12 calibrators were used. Similarly, for folic acid, VITROS folate reagent packs 1/2, VITROS vitamin B12/folate reagent pack 3 and VITROS folate calibrators were used.

#### Vitamin D

Using a competitive immunoassay technique, a low pH denaturant was used to release 25-OH vitamin D in the serum sample from its endogenous binding protein. The free 25-OH vitamin D competes with horseradish peroxidase (HRP)-labeled 25-OH vitamin D reagent for the monoclonal anti-vitamin D bound to the wells. The unbound materials were removed by washing.

#### Vitamin B12

Vitamin B12 present in the serum sample was released from its endogenous binding proteins by alkaline denaturation. Biotinylated Intrinsic Factor (IF) conjugate was then added and incubated with the neutralized sample. An aliquot of this sample was then transferred into a streptavidin-coated well and B12-HRP conjugate was added. A competitive binding reaction occurs, resulting in the vitamin B12-IF complexes being captured by streptavidin on the wells. The unbound materials were then removed by washing.

#### Folic Acid

Following alkaline denaturation (to release folate from its endogenous binding proteins) and stabilization (to prevent oxidation), an aliquot of the treated sample was transferred into a streptavidin-coated well. Folate-HRP and biotinylated folate binding protein (FBP) conjugate were added. Following a competitive binding reaction (folate in the serum sample competes with HRP-labeled folate for a limited number of binding sites on a biotinylated FBP), the FBP complexes are captured by streptavidin on the wells. Unbound conjugates were removed by washing.

The bound HRP conjugates for 25-OH vitamin D, B12 and folate were measured by a luminescent reaction (18). Reagent containing luminogenic substrates (luminol derivative and peracid salt) and an electron transfer agent (substituted acetanilide) was added to the wells. Oxidation of the luminol derivative was catalyzed by the HRP in the bound conjugates, thus producing light. The intensity and duration of light emission were enhanced by the electron transfer agent, and the light signals were read by the system. The amount of HRP conjugate bound is indirectly proportional to the concentration of 25-OH vitamin D, vitamin B12 and folate present in the sample, respectively.

For vitamin D, ≥30 ng/ml was considered as normal, <30 ng/ml was classified as low vitamin D and <20 ng/ml as vitamin D deficiency. For vitamin B12, ≥200 pg/ml was considered as normal and <200 pg/ml as deficiency. For folic acid, ≥3 ng/ml was considered as normal and <3 ng/ml as deficiency.

### Statistical Analysis

Statistical analysis was done using JASP open-source software, version 0.14.1. Primary quality assessment of range and consistency of the variables was done to check appropriateness of units and deviation from the population mean, to detect outliers. Age (45–54, 55–64, 65–74, ≥75 years) and gender distributions were tabulated. Frequency distributions of vitamin D, vitamin B12 and folic acid levels in the population were plotted. Mean levels of the above parameters were calculated. “t” test was performed to check for significant differences between mean values of males and females – both overall and in each of the age groups. ANOVA was done to compare the means of vitamin D, vitamin B12 and folic acid levels between all age groups and also, between age groups among females and males separately. Percentage of subjects having deficiency of the studied micronutrients was calculated according to gender as well as among different age-groups. Chi-squared test was done to check for any significant differences, gender-wise and age-group-wise. *P*-value of < 0.05 was considered as significant.

## Results

**Figure 1:**
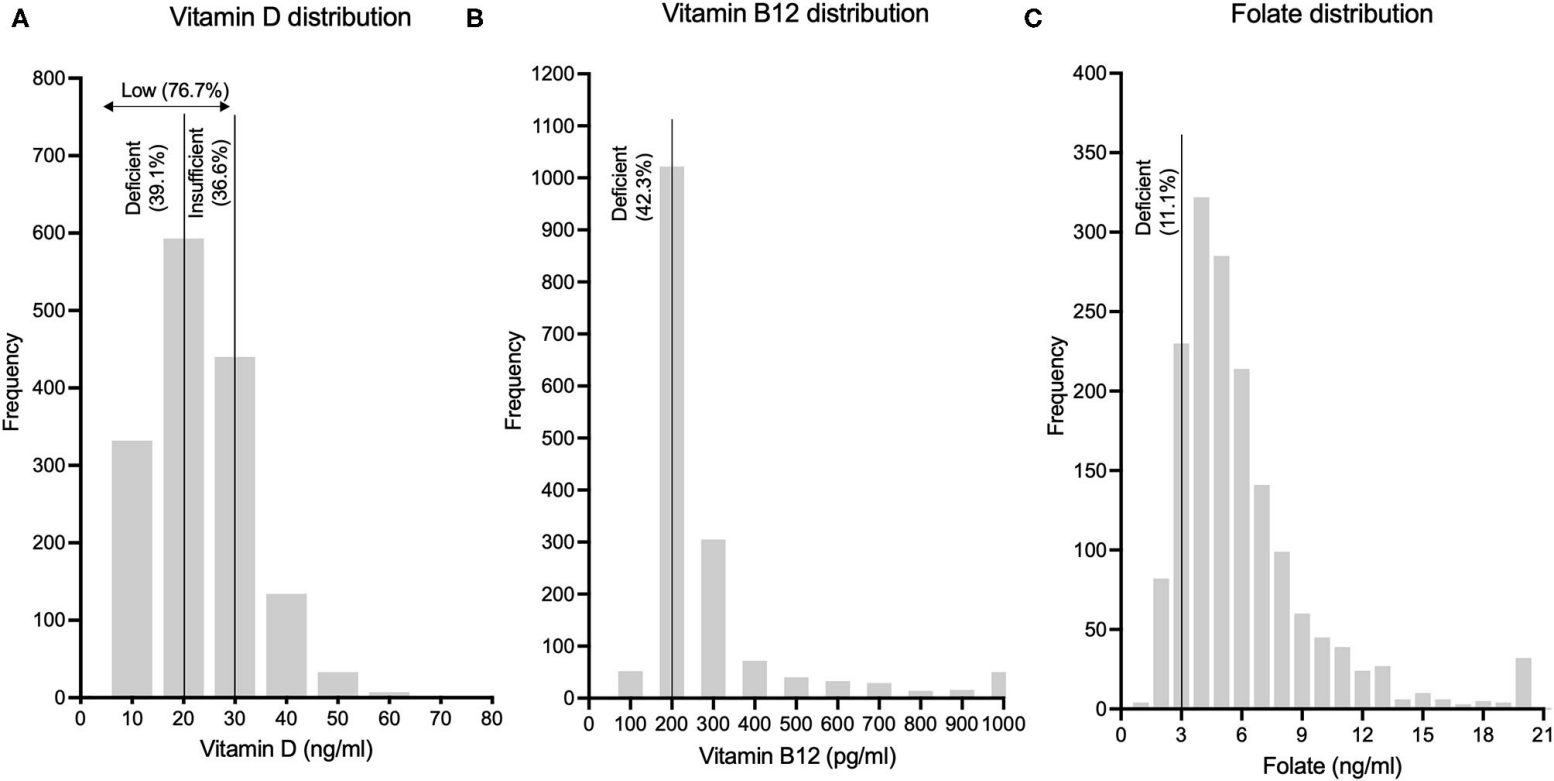
Frequency distributions of **(A)** Vitamin D levels, **(B)** Vitamin B12 levels and **(C)** Folic acid levels in this rural Indian population are depicted.

Gender distribution of the study population was 47.1% (*n* = 776) males and 52.9% females (*n* = 872). Mean age of the population was 58 ± 10.2 years, with males having a higher mean age than females (59.5 ± 10.4 vs. 56.6 ± 9.8 years). Out of the 1,648 subjects in this analytical sample, vitamin D levels were available for 1,546 subjects, vitamin B12 levels were available for 1,639 subjects and folic acid levels were available for 1,640 subjects. Frequency distribution of vitamin D, vitamin B12 and folic acid levels are shown in Figure 1. Mean levels of vitamin D, B12 and folic acid were 23.4 ± 10.6 ng/ml, 277.4 ± 194.4 pg/ml and 6 ± 3.5 ng/ml, respectively. Age and gender-stratified means with standard deviations for the above three parameters are represented in Table 1. Significant gender difference was observed in mean values for vitamin D (males > females; *p* < 0.001) and folic acid (females > males; *p* < 0.001) but not for vitamin B12. Significant difference was also seen in mean vitamin D among levels between different age groups – overall (*f* = 3.73, *p* = 0.011) as well as among males (*f* = 7.74, *p* < 0.001) but not among females (*f* = 2.01, *p* = 0.11). Tukey *post-hoc* test showed that there was significant difference in 55–64 years age group as compared to ≥75 years age group. Among males, this difference was significant in 45–54 years age group as compared to ≥75 years age group. Similarly, significant difference was also seen in 55–65 years age group as compared with 65–74 years and ≥75 years age groups among males. Significant difference was seen in vitamin B12 means between different age groups – overall (*f* = 4.99, *p* = 0.002) as well as among females (*f* = 5.68, *p* < 0.001) but not among males (*f* = 1.01, *p* = 0.38). On *post-hoc* analysis, significant difference was seen when the mean of 45–54 years age group was compared to 65–74 years and ≥75 years age groups. A similar difference was also observed between the same age groups among females. There was no statistically significant difference for the folate levels among different age groups, neither in the overall age groups nor in the gender-stratified age-groups.

**Table 1:** Age and gender-stratified mean levels of vitamin D, vitamin B12 and folic acid.

**Parameters**			**Age group (years)**	**Gender**		**Female vs. Male** **(** ***t*** **-test)**	
				**Female**	**Male**	***T***	***P***
**Vitamin D (ng/ml)**			45–54	21.60 (9.81)	25.65 (10.39)	−5.07	<0.001^*^
			55–64	20.69 (11.24)	24.19 (10.06)	−3.38	<0.001^*^
			65–74	20.65 (8.37)	27.62 (10.43)	−6.74	<0.001^*^
			≥75	18.21 (7.77)	30.34 (12.10)	−6.43	<0.001^*^
			Overall	20.94 (9.87)	26.21 (10.65)	−10.1	<0.001^*^
	**Between age**	***F***	3.73	2.01	7.74		
	**groups (ANOVA)**	***p***	0.011^*^	0.11	<0.001^*^		
**Vitamin B12 (pg/ml)**			45–54	252.20 (165.41)	269.90 (185.68)	−1.31	0.18
			55–64	287.95 (204.23)	267.74 (184.45)	1.11	0.26
			65–74	309.88 (212.41)	281.27 (196.93)	1.31	0.191
			≥75	335.39 (269.42)	307.90 (231.01)	0.59	0.55
			Overall	278.74 (195.59)	275.94 (193.20)	0.29	0.77
	**Between age**	***F***	4.99	5.68	1.012		
	**groups (ANOVA)**	***P***	0.002^*^	<0.001^*^	0.38		
**Folate (ng/ml)**			45–54	6.08 (3.08)	5.6 (3.04)	2.05	0.041^*^
			55–64	6.71 (4.10)	5.46 (3.11)	3.66	<0.001^*^
			65–74	6.68 (4.01)	5.98 (3.59)	1.73	0.08
			≥75	5.98 (4.18)	5.58 (3.62)	0.63	0.52
			Overall	6.37 (3.66)	5.65 (3.26)	4.2	<0.001^*^
	**Between age**	***F***	1.61	2.12	0.89		
	**groups (ANOVA)**	***P***	0.18	0.095	0.44		

*This table shows the mean levels of vitamin D, vitamin B12 and folic acid in a rural, aging cohort from southern India. The data is stratified according to gender (female and male) and age-groups (45–54, 55–64, 65–74, and ≥75 years). Statistical significance in the difference in mean values between females and males (calculated using t-test) and difference in mean values (calculated using ANOVA) is represented as p-values*.

* *p < 0.05 was considered as significant*.

**Figure 2:**
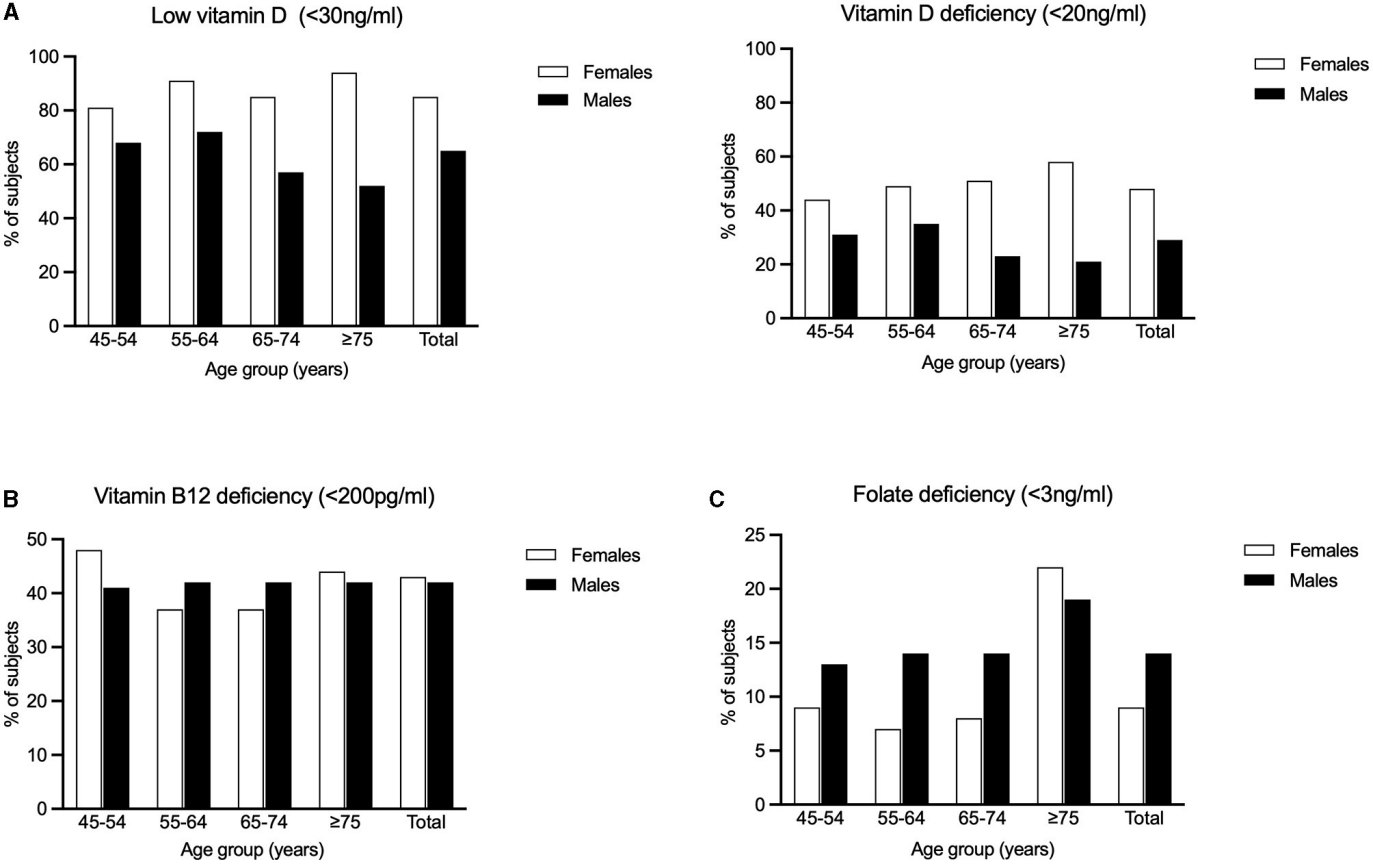
Percentages of the study population having **(A)** Low Vitamin D (<30 ng/ml) and Vitamin D deficiency (<20 ng/ml) **(B)** Vitamin B12 deficiency and **(C)** Folic acid deficiency is graphically represented.

We found that the overall prevalence of low vitamin D (<30 ng/ml) in our rural cohort was 75.7%, with significantly higher prevalence in females compared to males (85.5% vs. 64.7%; *p* < 0.001). On age stratification (45–54 years, 55–64 years, 65–74 years and ≥75 years), this gender difference (females > males) was significant (*p* < 0.001) across all the above-mentioned age groups. Females in the age group of 75 years and above had the highest prevalence (94.3%) of low vitamin D. The overall prevalence of vitamin D deficiency (<20 ng/ml) in our rural cohort was 39.1%, with significantly higher prevalence in females compared to males (47.8% vs. 29.2%; *p* < 0.001). The same trend was observed with statistical significance across all age groups: 45–54, 55–64, 65–74, and ≥75 years. The highest prevalence of vitamin D deficiency (59.6%) was seen in females in the age group of 75 years and above (Figure 2).

The overall prevalence of vitamin B12 deficiency (<200 pg/ml) in our rural subjects was 42.3%. There was no significant difference in prevalence between males and females (41.8% vs. 42.7%). However, among females, significantly higher prevalence was observed in the age group of 45–54 years (*p* = 0.014) There was no significant difference between age groups among males (Figure 2).

The overall prevalence of folic acid deficiency (<3 ng/ml) in our rural cohort was 11.1% There was significantly (*p* < 0.001) higher prevalence in males (13.9%) compared to females (8.6%). Among the different age groups, we found that subjects aged 75 years and above had the highest prevalence (19.9%, *p* < 0.001). In this oldest age group, females had a slightly higher prevalence compared to males (21.8% vs. 18.5%), though this difference was not statistically significant (Figure 2).

## Discussion

Our study aimed at determining the burden of vitamin D, vitamin B12 and folic acid deficiencies in an aging (≥45 years), rural community from the state of Karnataka in southern India. Our results revealed that the overall prevalence of low vitamin D (<30 ng/ml), vitamin D deficiency (<20 ng/ml), vitamin B12 deficiency (<200 pg/ml) and folic acid deficiency (<3 ng/ml) were 75.7%, 39.1%, 42.3%, and 11.1%, respectively. These results imply that this rural community had an overall higher burden of inadequate vitamins D and B12, with relatively low burden of folic acid deficiency.

The definition of adequate levels of vitamin D has undergone much debate in the recent past, with recommendations varying between different expert advisory bodies. For example, the US National Academy of Medicine (formerly Institute of Medicine) (19) recommends levels of 20 ng/ml and above as adequate, whereas the US Endocrine Society (20) and International Osteoporosis Foundation (21) recommendations classify levels of 20–29 ng/ml as “insufficient” and <20 ng/ml as “deficient.” In our study, we used two cut-off levels to categorize abnormal levels: <30 ng/ml was categorized as low vitamin D (to include both insufficiency and deficiency) and <20 ng/ml was categorized as vitamin D deficiency.

Relatively less studies on vitamin D deficiency have been carried out in tropical countries as compared to temperate countries (22). Moreover, in the background of scarce information on vitamin D status of Indians in rural communities, our finding that over three-fourths of this rural Indian population had low vitamin D is very concerning. This also goes against the traditional view that individuals from tropical countries, which receive sunshine throughout the year, are likely to have adequate levels of vitamin D. Possible reasons for the particularly high prevalence in this cohort are older population having minimal outdoor activity, increasing modernization and usage of machines for agricultural work (thus, decreasing manual labor in the open fields) and poor dietary source of vitamin D, since most individuals have no or minimal intake of meat products and milk is not uniformly fortified across the country.

The burden of vitamin D deficiency in our study (39.1%) is similar to that among European (40.4%) (23) and American (41.6%) (24) adults. Studies from India in adult populations have shown wide variations, though some studies (25, 26) have shown significantly higher prevalence in urban compared to rural areas. This trend was also observed when we compared our rural study results with unpublished results from our parallel, harmonized, urban, aging cohort from Bangalore city in India (the rural and urban study sites are roughly 60 miles apart within the same state). In this urban cohort (Tata Longitudinal Study of Aging, TLSA), overall prevalence of vitamin D deficiency was found to be much higher (urban, TLSA cohort – 61.5% vs. rural, SANSCOG cohort – 39.1%). Another recent study in an urban, aging community from Delhi in northern India found the prevalence of vitamin D deficiency to be as high as 91.2% (27). This difference in rural as compared to urban Indian populations could be because rural-dwelling residents, who are mostly engaged in agricultural work in the fields get more exposure to sunlight, which is protective against vitamin D deficiency.

Our study also revealed significant gender difference, with higher prevalence of vitamin D deficiency in women compared to men (64.7% vs. 29.2%), which has been observed in some (26, 28) but not in other studies from India (27, 29). One possible reason for this could be that higher proportion of men as compared to women are engaged in outdoor agricultural work in rural areas. The extremely high prevalence (94.3%) of low vitamin D in elderly women aged 75 years and above in our study is disturbing, since these individuals are more vulnerable for cognitive decline, and inadequate levels of vitamin D could further worsen cognitive ability. With recent studies (30) showing that low vitamin D is associated with higher risk of subsequent cognitive decline in elderly, the above results could have major implications in formulating preventive or interventional strategies for cognitive disorders in elderly. Low vitamin D status has also been found to be significantly associated with the risk of frailty (31), particularly in older women (32). There is also emerging evidence that vitamin D deficiency is associated with adverse mood changes and depression in the elderly (7, 33). Further, both frailty and depression have been shown to be predictors of cognitive decline among elderly (34, 35). The multifaceted role of vitamin D extends to maintaining healthy immune function (36, 37). Increased levels of serum inflammatory markers have been found in healthy persons with vitamin D insufficiency (38) and more recently, the association of vitamin D status with viral infections including COVID-19 is coming to light (39, 40). This is important since the accumulating evidence points to the role of the immune system and inflammation in Alzheimer's disease and related dementias (41, 42).

Compared to the scarce literature on vitamin D deficiency, studies on vitamin B12 and folic acid from India are relatively higher. However, majority of these studies have focussed on young adults, especially, women in the reproductive age-group and nation-wide data is lacking (43). Sub-optimal levels of Vitamin B12 has been reported to be common in less developed countries (44) as well as among the elderly (45). The overall prevalence of vitamin B12 deficiency (42.3%) in our study is much higher compared to literature from Western countries (46, 47), including studies in older populations (48). However, it closely compares with that reported in two recent studies [35% (49) and 46% (50)] from an urban setting in southern India. Another recent study (51) from northern India reported a prevalence of 47.19%. All the above-mentioned Indian studies were carried out in adults and not specifically in the aging population. A small study (52) from Bangalore, southern India reported the prevalence of vitamin B12 deficiency (<148 pmol/L) to be significantly higher in the elderly age group (>60 years, 61.7%, *n* = 47) as compared to young adults (20–40 years, 56.2%, *n* = 32). Vitamin B12 deficiency, particularly in older adults is a matter of concern since low levels of B12 have been associated with poorer cognitive performance in specific cognitive domains, as revealed by a recent large, cohort study in UK (53).

Vegetarian diet is known to be associated with higher prevalence of vitamin B12 deficiency in India (54). In our rural cohort, majority of participants follow a vegetarian or ovo-vegetarian diet. Though a minor proportion consume meat, the frequency of consumption is low (usually once or twice a month). Hence, their dietary habits could be a significant contributor to the high prevalence of vitamin B12 deficiency. Moreover, the use of vitamin supplements was not observed in this rural population as compared to our urban (TLSA) cohort, where we observed that the use of supplements was common, and prevalence of vitamin B12 deficiency was lower (30.7%, unpublished data).

Overall prevalence of folic acid deficiency (11.1%) in our study is comparable to that reported in a recent study (49) conducted in an urban community of apparently healthy adults from southern India (12%). However, in contrast to this study, which showed no significant difference in folic acid levels between different age groups, our study showed a significantly higher prevalence in the age group of ≥ 75 years. Another small study (55) among a sample of 60 deprived elderly women aged 60–70 years from New Delhi in India showed a similar prevalence of folate deficiency (using the cut-off <10 nmol/L). A recent study (56) on a geriatric, rural Indian population revealed that 72% of subjects (aged 60 years and above) did not consume the recommended dietary allowance (RDA) of folic acid (400 μg/day). On the other hand, a study (57) on elderly subjects (aged 60 years and above) from urban India showed that 51% consumed less than the RDA of folic acid. Though previous studies from India have highlighted folic acid deficiency in the adolescent (58) and peri-conceptional age groups (59), ours is one of the very few studies that highlight significant deficiency in the elderly age group. This is important given the association of folate deficiency with depression and dementia in this geriatric age group (60).

Key strengths of our study include a large, homogenous, community-based, rural sample of aging individuals, with bioassays performed in a reputed, nationally accredited laboratory. Limitations include non-representative sampling, and thus, these results cannot be generalized to the whole of India. Following up this cohort of participants, along with periodic monitoring of their cognitive status can give valuable insights to understand whether these micronutrient deficiencies are causally associated with cognitive impairment and other aging-related disorders.

Owing to the significant burden of these vitamin deficiencies in India, well-structured, community-level strategies, including vitamin supplementation or food fortification, need to be implemented at the state or national level. The burden thus straddles generations and has public health implications now and also, into the future. India has programs that target children (megadose vitamin A supplementation for children under 6 years of age) and pregnant women (folic acid supplementation), however, none for the elderly. Recent prospective trials on vitamin D fortification of milk among children and adolescents in India (61, 62) have shown that this is a safe and effective measure to address vitamin D deficiency. However, it must be taken into account that the usual quantities of milk or other dairy products consumed by individuals belonging to marginalized, rural communities are minimal, and hence, dairy fortification may not fully serve its purpose in such situations, particularly amongst the elderly. Home fortification of staple foods, such as cereal flours (rice, wheat, ragi, etc.) using multiple micronutrient powders could be a feasible alternative. Moreover, this would be culturally acceptable and easily implementable. For this to happen, it is vital to massively ramp up health-related awareness among the rural elderly and envision continual, sustainable strategies which inculcate the significance of vitamin deficiencies and also, their adverse impact on multiple health outcomes.

## Data Availability Statement

The raw data supporting the conclusions of this article will be made available by the authors, without undue reservation.

## Ethics Statement

The studies involving human participants were reviewed and approved by Centre for Brain Research (CBR) - Institutional Ethics Committee. The patients/participants provided their written informed consent to participate in this study.

## Author's Note

We reviewed existing literature on the prevalence of micronutrient deficiencies among the older population in India and found that the current evidence, especially from rural India, is scarce. Vitamin D, B12 and folic acid deficiencies in older adults can potentially have an adverse impact on aging and have been implicated in aging-related disorders including dementia. Therefore, we wanted to ascertain the burden of these deficiencies in a rural, aging community in India. We conducted this study in an aging cohort of community-dwelling individuals from villages of Srinivaspura in southern India. Our study revealed that rural-dwelling, older Indians had high burden of vitamin D and B12 deficiencies and relatively low burden of folic acid deficiency. The burden of certain deficiencies was higher in women and among the oldest age groups. Our findings bring to light that micronutrient deficiencies are a major public health concern in rural India and can help inform public health policies to prevent or mitigate their adverse consequences. We plan to follow-up our subjects long-term, particularly focusing on monitoring of cognitive changes as they age. This will give us unparalleled insights into the possible causal association of these vitamin deficiencies with aging-related, cognitive disorders, such as dementia.

## Author Contributions

JS, SS, and VR: have made a substantial intellectual contribution to the conception and design or conduct of the study. JS and VR: analysis and interpretation of data, writing, and reviewing the manuscript. SS: acquisition of data, statistical analysis, drafting figures, and reviewing the manuscript. All authors contributed to the article and approved the submitted version.

## SANSCOG Study Team

BN Gangadhar, MD, NIMHANS^1^, Psychiatry, kalyanybg@yahoo.com; Bratati Kahali, PhD, CBR, IISc^2^, Computational Genetics and Bioinformatics, bratati@iisc.ac.in; Girish N Rao, MD, NIMHANS^1^, Public Health and Epidemiology, girishnrao@yahoo.com; Kalyani Raju, MD, SDUAHER^3^, Pathology, drkalyanir@rediffmail.com; Naren P Rao, MD, NIMHANS^1^, Psychiatry, docnaren@gmail.com; Palanimuthu T Sivakumar, MD, NIMHANS^1^, Psychiatry, sivakumar.nimhans@gmail.com; Smitha Karunakaran, PhD, CBR, IISc^2^, Neural circuit mechanisms underlying dementia, smitha@iisc.ac.in; Vivek Tiwari, PhD, CBR, IISc^2^, Magnetic Resonance Imaging, vivekt@iisc.ac.in.

^1^National Institute of Mental Health and Neurosciences, Bangalore, India.

^2^Centre for Brain Research, Indian Institute of Science, Bangalore, India.

^3^Sri Devaraj Urs Academy of Higher Education and Research, Kolar, India.

## Conflict of Interest

The authors declare that the research was conducted in the absence of any commercial or financial relationships that could be construed as a potential conflict of interest.

## Publisher's Note

All claims expressed in this article are solely those of the authors and do not necessarily represent those of their affiliated organizations, or those of the publisher, the editors and the reviewers. Any product that may be evaluated in this article, or claim that may be made by its manufacturer, is not guaranteed or endorsed by the publisher.

## References

[1] Sassi F Tamone C D'amelio P. Vitamin D: nutrient, hormone, and immunomodulator. Nutrients. (2018) 10:1656. 10.3390/nu10111656 30400332 PMC6266123

[2] Holick MF. Medical progress: Vitamin D deficiency. N Engl J Med. (2007) 357:266–81. 10.1056/NEJMra070553 17634462

[3] Ryan-Harshman M Aldoori W. Vitamin B12 and health. Can Family Phys. (2008) 54:536–41. PMC2294088 18411381

[4] Stover PJ. Physiology of folate and vitamin B12 in health and disease. Nutr Rev. (2004) 62:S3–12. 10.1111/j.1753-4887.2004.tb00070.x 15298442

[5] Holick MF. The vitamin D epidemic and its health consequences. J Nutr. (2005) 135:2739S–48S. 10.1093/jn/135.11.2739S 16251641

[6] Bouillon R Marcocci C Carmeliet G Bikle D White JH Dawson-Hughes B. Skeletal and extraskeletal actions of vitamin D: current evidence and outstanding questions. Endoc Rev. (2019) 40:1109–51. 10.1210/er.2018-00126 30321335 PMC6626501

[7] Barnard K Colón-Emeric C. Extraskeletal effects of vitamin D in older adults: cardiovascular disease, mortality, mood, and cognition. Am J Geriatr Pharmacother. (2010) 8:4–33. 10.1016/j.amjopharm.2010.02.004 20226390

[8] O'Leary F Samman S. Vitamin B12 in health and disease. Nutrients. (2010) 2:299. 10.3390/nu2030299 22254022 PMC3257642

[9] Molloy AM Kirke PN Brody LC Scott JM Mills JL. Effects of folate and vitamin B12 deficiencies during pregnancy on fetal, infant, and child development. Food Nutr Bull. (2008) 29(2 SUPPL.):S101–11. 10.1177/15648265080292S114 18709885

[10] Kronenberg G Colla M Endres M. Folic acid, neurodegenerative and neuropsychiatric disease. Curr Mol Med. (2009) 9:315–23. 10.2174/156652409787847146 19355913

[11] Meehan M Penckofer S. The role of vitamin D in the aging adult. J Aging Gerontol. (2014) 2:60–71. 10.12974/2309-6128.2014.02.02.1 25893188 PMC4399494

[12] Goodwill AM Szoeke C. A systematic review and meta-analysis of the effect of low vitamin d on cognition. J Am Geriatr Soc. (2017) 65:2161–8. 10.1111/jgs.15012 28758188

[13] Smith AD Refsum H. Vitamin B-12 and cognition in the elderly. Am J Clin Nutr. (2009) 89:707S–11S. 10.3945/ajcn.2008.26947D 19116332

[14] Rampersaud GC Kauwell GPA Bailey LB. Folate: a key to optimizing health and reducing disease risk in the elderly. J Am Coll Nutr. (2003) 22:1–8. 10.1080/07315724.2003.10719270 12569109

[15] Malouf R Evans JG. Folic acid with or without vitamin B12 for the prevention and treatment of healthy elderly and demented people. Cochrane Database Syst Rev. (2008). CD004514. 10.1002/14651858.CD004514.pub2 18843658 PMC12926861

[16] Sundarakumar J Chauhan G Rao GN Sivakumar PT Rao NP Ravindranath V. Srinivaspura Aging, Neuro Senescence and COGnition (SANSCOG) study and Tata Longitudinal Study on Aging (TLSA): Study protocols. Alzheimers Dement. (2020) 16(Suppl. 4):e045681. 10.1002/alz.045681

[17] Lai JKC Lucas RM Clements MS Harrison SL Banks E. Assessing vitamin D status: pitfalls for the unwary. Mol Nutr Food Res. (2010) 54:1062–71. 10.1002/mnfr.200900468 20397196

[18] Summers M Booth T Brockas T Edgar H Edwards J Nunnerley C. Luminogenic reagent using 3-chloro-4-hydroxy acetanilide to enhance peroxidase luminol chemiluminescence. Clin Chem. (1995) 41:S73.

[19] Ross AC Manson JAE Abrams SA Aloia JF Brannon PM Clinton SK. The 2011 report on dietary reference intakes for calcium and vitamin D from the institute of medicine: what clinicians need to know. J Clin Endocrinol Metab. (2011) 96:53–58. 10.1210/jc.2010-2704 21118827 PMC3046611

[20] Holick MF Binkley NC Bischoff-Ferrari HA Gordon CM Hanley DA Heaney RP. Evaluation, treatment, and prevention of vitamin D deficiency: an endocrine society clinical practice guideline. J Clin Endocrinol Metab. (2011) 96:1911–30. 10.1210/jc.2011-0385 21646368

[21] Dawson-Hughes B Mithal A Bonjour JP Boonen S Burckhardt P Fuleihan GEH. IOF position statement: Vitamin D recommendations for older adults. Osteopor Int. (2010) 21:1151–4. 10.1007/s00198-010-1285-3 20422154

[22] Wahl DA Cooper C Ebeling PR Eggersdorfer M Hilger J Hoffmann K. A global representation of vitamin D status in healthy populations. Arch Osteopor. (2012) 7:155–72. 10.1007/s11657-012-0093-0 23225293

[23] Cashman KD Dowling KG Škrabáková Z Gonzalez-Gross M Valtueña J De Henauw S. Vitamin D deficiency in Europe: Pandemic? Am J Clin Nutr. (2016) 103:1033–44. 10.3945/ajcn.115.120873 26864360 PMC5527850

[24] Forrest KYZ Stuhldreher WL. Prevalence and correlates of vitamin D deficiency in US adults. Nutr Res. (2011) 31:48–54. 10.1016/j.nutres.2010.12.001 21310306

[25] Harinarayan CV Ramalakshmi T Venkataprasad U. High prevalence of low dietary calcium and low vitamin D status in healthy south Indians. Asia Pac J Clin Nutr. (2004) 13:359–64. 15563441

[26] Harinarayan CV Ramalakshmi T Prasad UV Sudhakar D. Vitamin D status in Andhra Pradesh: A population based study. Indian J Med Res. (2008) 127:211–8. 18497434

[27] Marwaha RK Tandon N Garg MK Kanwar R Narang A Sastry A. Vitamin D status in healthy Indians aged 50 years and above. J Assoc Physicians India. (2011) 59:706–9. 22616336

[28] Harinarayan CV Ramalakshmi T Prasad UV Sudhakar D Srinivasarao PVLN. High prevalence of low dietary calcium, high phytate consumption, and vitamin D deficiency in healthy south Indians. Am J Clin Nutr. (2007) 85:1062–7. 10.1093/ajcn/85.4.1062 17413106

[29] Suryanarayana P Arlappa N Sai Santhosh V Balakrishna N Lakshmi Rajkumar P Prasad U. Prevalence of vitamin D deficiency and its associated factors among the urban elderly population in Hyderabad metropolitan city, South India. Ann Hum Biol. (2018) 45:133–9. 10.1080/03014460.2018.1425479 29307228

[30] Llewellyn DJ Lang IA Langa KM Muniz-Terrera G Phillips CL Cherubini A. Vitamin D and risk of cognitive decline in elderly persons. Arch Int Med. (2010) 170:1135–41. 10.1001/archinternmed.2010.173 20625021 PMC4053858

[31] Bruyere O Cavalier E Buckinx F Reginster JY. Relevance of Vitamin D in the pathogenesis and therapy of frailty. Curr Opin Clin Nutri Metab Care. (2017) 20:26–9. 10.1097/MCO.0000000000000334 27749712

[32] Buchebner D Bartosch P Malmgren L McGuigan FE Gerdhem P Akesson KE. Association between Vitamin D. Frailty, and progression of frailty in community-dwelling older women. J Clin Endocrinol Metab. (2019) 104:6139–47. 10.1210/jc.2019-00573 31287540

[33] Anglin RES Samaan Z Walter SD Sarah DM. Vitamin D deficiency and depression in adults: systematic review and meta-analysis. Bri J Psychiatry. (2013) 202:100–7. 10.1192/bjp.bp.111.106666 23377209

[34] Borges MK Canevelli M Cesari M Aprahamian I. Frailty as a predictor of cognitive disorders: a systematic review and meta-analysis. Front Med. (2019) 6:26. 10.3389/fmed.2019.00026 30838210 PMC6389599

[35] Norton S Matthews FE Barnes DE Yaffe K Brayne C. Potential for primary prevention of Alzheimer's disease: an analysis of population-based data. Lancet Neurol. (2014) 13:788–94. 10.1016/S1474-4422(14)70136-X 25030513

[36] Aranow C. Vitamin D and the immune system. J Invest Med. (2011) 59:881–6. 10.2310/JIM.0b013e31821b8755 21527855 PMC3166406

[37] Peelen E Knippenberg S Muris AH Thewissen M Smolders J Tervaert JWC. Effects of vitamin D on the peripheral adaptive immune system: a review. Auto Rev. (2011) 10: 733–43. 10.1016/j.autrev.2011.05.002 21621002

[38] Peterson CA Heffernan ME. Serum tumor necrosis factor-alpha concentrations are negatively correlated with serum 25(OH)D concentrations in healthy women. J Inflamm. (2008) 5:10. 10.1186/1476-9255-5-10 18652680 PMC2503979

[39] Rhodes JM Subramanian S Laird E Kenny RA. Editorial: Low population mortality from COVID-19 in countries south of latitude 35 degrees North supports vitamin D as a factor determining severity. Aliment Pharmacol Ther. (2020) 51:1434–7. 10.1111/apt.15777 32311755 PMC7264531

[40] Calder P Carr A Gombart A Eggersdorfer M. Optimal nutritional status for a well-functioning immune system is an important factor to protect against viral infections. Nutrients. (2020) 12:1181. 32756516 10.3390/nu12082326 PMC7469053

[41] Van Eldik LJ Carrillo MC Cole PE Feuerbach D Greenberg BD Hendrix JA. The roles of inflammation and immune mechanisms in Alzheimer's disease. Alzheimers Dement. (2016) 2:99–109. 10.1016/j.trci.2016.05.001 29067297 PMC5644267

[42] Cao W Zheng H. Peripheral immune system in aging and Alzheimer's disease. Mol. Neurodegener. (2018) 13:51. 10.1186/s13024-018-0284-2 30285785 PMC6169078

[43] Gonmei Z Toteja GS. Micronutrient status of Indian population. Indian J Med Res. (2018) 148:511–21. 10.4103/ijmr.IJMR_1768_1810.4103/ijmr.IJMR_1768_18 30666978 PMC6366258

[44] Smith AD Warren MJ Refsum H. Vitamin B12. Adva Food Nutr Res. (2018) 83:215–79. 10.1016/bs.afnr.2017.11.005 29477223

[45] Allen RH Lindenbaum J Stabler SP. High prevalence of cobalamin deficiency in the elderly. Trans Am Clin Climatol Assoc. (1996) 107:37–47. 8725558 PMC2376575

[46] Hunt A Harrington D Robinson S. Vitamin B12 deficiency. BMJ. (2014) 349:g5226. 10.1136/bmj.g5226 25189324

[47] Mineva E Sternberg M Pfeiffer CM. Prevalence of vitamin B-12 deficiency in US adults using the combined indicator of vitamin B-12 status, cB12. Curr Dev Nutr. (2020) 4(Suppl. 2):1824. 10.1093/cdn/nzaa067_051

[48] O'Connor DMA Laird EJ Carey D O'Halloran AM Clarke R Kenny RA. Plasma concentrations of vitamin B 12 and folate and global cognitive function in an older population: cross-sectional findings from the irish longitudinal study on ageing (TILDA). Bri J Nutr. (2020) 124:602–10. 10.1017/S0007114520001427 32329423

[49] Sivaprasad M Shalini T Balakrishna N Sudarshan M Lopamudra P Suryanarayana P. Status of vitamin B12 and folate among the urban adult population in South India. Ann Nutr Metab. (2016) 68:94–102. 10.1159/000442677 26667891

[50] Sivaprasad M Shalini T Reddy PY Seshacharyulu M Madhavi G Kumar BN. Prevalence of vitamin deficiencies in an apparently healthy urban adult population: assessed by subclinical status and dietary intakes. Nutrition. (2019) 63–64, 106–113. 10.1016/j.nut.2019.01.017 30954757

[51] Singla R Garg A Surana V Aggarwal S Gupta G Singla S. Vitamin B12 deficiency is endemic in Indian population: a perspective from North India. Indian J Endocrinol Metab. (2019) 23:211. 10.4103/ijem.IJEM_122_19 31161105 PMC6540890

[52] Sucharita S Thomas T Sowmya S Krishnamachari S Kurpad VA Vaz M. Subclinical vitamin B12 deficiency and heart rate variability across life cycle. Curr Aging Sci. (2016) 9:217–23. 10.2174/1874609809666160211125218 26864037

[53] NalderLZhengBChiandetGMiddletonLTde JagerCA. Vitamin B12 and folate status in cognitively healthy older adults and associations with cognitive performance. J Nutr Health Aging. (2021) 25:287–94. 10.1007/s12603-020-1489-y33575718

[54] Pawlak R Parrott SJ Raj S Cullum-Dugan D Lucus D. How prevalent is vitamin B12 deficiency among vegetarians? Nutr Rev. (2013) 71:110–7. 10.1111/nure.12001 23356638

[55] Gonmei Z Siddhu A Toteja GS Dwivedi S Chand V Vikram NK. Hemoglobin, folate and vitamin B12 status of economically deprived elderly women. J Clin Gerontol Geriatr. (2017). 8:133–4. 10.24816/jcgg.2017.v8i4.06

[56] Gupta A Ramakrishnan L Pandey R Sati H Khandelwal R Khenduja P. Risk factors of anemia amongst elderly population living at high-altitude region of India. J Fam Med Prim Care. (2020) 9:673. 10.4103/jfmpc.jfmpc_468_19 32318402 PMC7113975

[57] Shobha V Tarey SD Singh RG Shetty P Unni US Srinivasan K. Vitamin B12 deficiency & levels of metabolites in an apparently normal urban south Indian elderly population. Indian J Med Res. (2011) 134:432–9. 22089603 PMC3237239

[58] Kapil U Bhadoria A. Prevalence of folate, ferritin and cobalamin deficiencies amongst adolescent in India. J Fam Med Prim Care. (2014) 3:247. 10.4103/2249-4863.141619 25374863 PMC4209681

[59] Bhide P Kar A. Prevalence and determinants of folate deficiency among urban Indian women in the periconception period. Eur J Clin Nutr. (2019) 73:1639–41. 10.1038/s41430-018-0255-2 30026502

[60] Reynolds EH. Folic acid, ageing, depression, and dementia. Bri Med J. (2002) 324:1512–5. 10.1136/bmj.324.7352.1512 12077044 PMC1123448

[61] Khadgawat R Marwaha RK Garg MK Ramot R Oberoi AK Sreenivas V. Impact of vitamin D fortified milk supplementation on vitamin D status of healthy school children aged 10-14 years. Osteop Int. (2013) 24:2335–43. 10.1007/s00198-013-2306-9 23460234

[62] Garg MK Marwaha RK Khadgawat R Ramot R Obroi AK Mehan N. Efficacy of vitamin D loading doses on serum 25-hydroxy vitamin D levels in school going adolescents: an open label non-randomized prospective trial. J Pediatr Endocrinol Metab. (2013) 26:515–23. 10.1515/jpem-2012-0390 23509211

